# Biphasic anaphylaxis in a Canadian tertiary care centre: an evaluation of incidence and risk factors from electronic health records and telephone interviews

**DOI:** 10.1186/s13223-024-00919-2

**Published:** 2025-02-08

**Authors:** Anne K. Ellis, Lubnaa Hossenbaccus, Sophia Linton, Hannah Botting, Eman Badawod, Alyssa Burrows, Sarah Garvey

**Affiliations:** 1 Department of Medicine, Queen’s University, Kingston, ON Canada; 2 Allergy Research Unit, Kingston Health Sciences Center, KGH Site, Kingston, ON Canada; 3 Clinical Immunology and Allergy Division, Internal Medicine Department, King Abdulaziz University, Jeddah, Saudi Arabia; 4 Kingston Health Science Centre, Kingston General Hospital, Watkins 1D, 76 Stuart Street, Kingston, ON K7L 2V7 Canada

**Keywords:** Anaphylaxis, Biphasic anaphylaxis, Allergic reaction, Tertiary care centre, Tertiary care center, Hypersensitivity, Epinephrine, Food allergy, Drug allergy, Insect sting allergy

## Abstract

**Background:**

Our previous 2007 study reported a 19.4% rate of biphasic anaphylaxis in Kingston, Ontario. Since then, few updates have been published regarding the etiology and risk factors of biphasic anaphylaxis. This study aimed to describe the incidence of and predictors of biphasic anaphylaxis in a single centre through a retrospective evaluation of patients with diagnosed anaphylaxis.

**Methods:**

From November 2015 to August 2017, all patients who presented to the emergency department at two hospital sites in Kingston given a diagnosis of “allergic reaction,” “anaphylaxis,” “drug allergy,” or “insect sting allergy,” were evaluated. Patients were contacted sometime after ED discharge to obtain consent and confirm symptoms and timing of the reaction. A trained allergist determined if criteria for anaphylaxis were met and categorized the reactions as being uniphasic, biphasic, or non-anaphylactic biphasic. A full medical review of the event ensued, and each type of anaphylactic event was statistically compared.

**Results:**

Of 138 anaphylactic events identified, 15.94% were biphasic reactions, 79.0% were uniphasic, and 5.07% were classified alternatively as a non-anaphylactic biphasic reaction. The average time of a second reaction was 19.0 h in patients experiencing biphasic reactivity. For biphasic anaphylaxis, the symptom profiles of second reactions were significantly less severe (p = 0.0002) compared with the initial reaction but significantly more severe than non-anaphylactic biphasic events (p < 0.0001).No differences of management were identified between events.

**Conclusion:**

The incidence of biphasic reactions in this cohort was 15.94% and the average second-phase onset was 19.0 h. In biphasic reactivity, it appears that the symptom profile second reaction is less severe compared to the first reaction.

## Background

Anaphylaxis is an acute, potentially life-threatening systemic allergic reaction. It is defined as “a serious allergic reaction that is rapid in onset and may cause death” [1]. Many triggers have been identified to cause anaphylaxis, including food, insect stings and medications [2]. The prevalence of anaphylaxis ranges from 0.05 to 3%, with increasing rates of hospitalizations. In the United States, rates of anaphylaxis hospitalizations increased from 20 to 25.1 per million population between 1999 and 2009 [3, 4]. Despite the increasing rates of hospitalizations, fatality rates remain relatively unchanged and rare, constituting less than 1% of total mortality risk [5, 6]. The severity of clinical presentation varies widely. Still, it involves the acute onset of illness and frequently involves the skin and/or mucosa and either respiratory compromise or low blood pressure or end-organ dysfunction [7]. The time course for anaphylaxis can be classified as uniphasic, where a patient experiences a single anaphylactic reaction; protracted, where a patient experiences a single anaphylactic reaction but symptoms persist for a longer duration despite treatment; or biphasic [8]. Standard practice recommendations are observing patients until signs and symptoms have resolved. If the risk for biphasic reactivity or anaphylaxis fatality is determined to be higher, then extended observation for a 6 h or more is appropriate [2]. Furthermore, the World Allergy Organization (WAO) anaphylaxis guidelines are valuable when considering discharge and follow-up among patients treated for anaphylaxis [9]. The WAO recommends that at the time of discharge, patients at risk of another episode of anaphylaxis should be prescribed and taught about self-administration of epinephrine and have a written personalized anaphylaxis emergency action plan and medication identification method [9].

According to the anaphylaxis: a 2023 practice parameter update, biphasic anaphylactic reactions are defined as the recurrence of a reaction or the development of new symptoms 1–48 h after the resolution of the initial presentation without further exposure to the trigger [10]. It is reported to occur in 1–20% of patients [11, 12]. The exact etiology for biphasic reactions remains unclear; however, some known risk factors include a more severe initial presentation of anaphylaxis, necessitating repeated epinephrine doses, wide pulse pressure, unknown anaphylaxis triggers, and cutaneous signs and symptoms [8, 12]. Delayed or underused epinephrine treatment for initial anaphylaxis has been identified as a risk factor for developing biphasic anaphylaxis [13–15]. Rapid and complete resolution of acute symptoms has also been related to a decreased risk of developing biphasic reactions [16, 17]. The mainstay of treatment for anaphylaxis is the rapid administration of intramuscular epinephrine. Other important management strategies include supine positioning, supplemental oxygen, and intravenous fluids. Second-line optional treatments include antihistamines, corticosteroids, and bronchodilators, but these agents should never be used in place of epinephrine to manage anaphylaxis [9].

In 2007, we reported an incidence rate of 19% (n = 20) for biphasic reactions where the mean time to onset of the second phase was 10 h (range 2–38 h) in Kingston, Ontario. In 2007, 70% of patients treated for anaphylaxis in the ED received epinephrine [17]. This study aims to provide an update on the incidence of, and potential predictors for, biphasic anaphylaxis in a single centre through a retrospective evaluation of patients with diagnosed anaphylaxis. While this examination of biphasic anaphylaxis in Kingston and its surrounding area involves a small number of patients, it nonetheless provides important insights into an understudied and underreported phenomenon.

## Methods

### Study Design and Time Period

The Queen’s University and Affiliated Teaching Hospitals Health Sciences Human Research Ethics Board reviewed this study and granted ethical clearance.

All patients with ED visits who were diagnosed with “allergic reaction,” “anaphylaxis,” “drug allergy,” or “insect sting allergy” from November 2015 to August 2017 were evaluated. We first received verbal consent from patients on the phone to complete a survey. Trained study staff reviewed medical records and discussed the allergic event with the participant within two months of occurrence. Using this hybrid data collection method ensured a comprehensive understanding of the allergic event, including the cause and timing of the reaction, symptoms, and any treatment received. Participants were questioned about common allergic symptoms using easy-to-understand language, and the ED records were also consulted, especially when evaluating clinically identifiable symptoms (e.g., tachycardia, hypoxemia). Initial interest in treatment utilized in the ED focused on the collection of data on the progression and sequence of treatment, including the class of treatment. Special attention was given to any symptoms reoccurring after the allergic reaction had completely cleared. After completion, we sent out consent forms and asked them to be signed and returned for documentation purposes, though it was not required for study participation. Patients’ medical records related to the incident were reviewed by a trained allergist, and cases were classified as uniphasic, biphasic, or non-anaphylactic biphasic reactions.

### Outcome Measures

Anaphylaxis was defined according to the Canadian Pediatric Surveillance Program (CPSP) as a severe allergic reaction to any stimulus, having sudden onset and generally lasting less than 24 h, involving at least two body systems, with multiple symptoms such as hives, flushing, angioedema, stridor, wheezing, shortness of breath, vomiting, diarrhea, or shock [17]. For a reaction to be labelled “biphasic,” the second-phase reaction must meet the same definition. Recurrence of urticaria or another rash alone was insufficient to qualify as a biphasic reaction. Following the complete resolution of anaphylaxis symptoms, several participants reported the recurrence of single-system reactions (e.g., urticaria alone). For this analysis, the descriptor “non-anaphylactic biphasic reaction” was assigned.

### Data Analysis

Statistical analyses were completed using GraphPad Prism 9.

## Results

From November 2015 to August 2017, 155 anaphylaxis events were identified, consisting of 148 unique patients, of whom 17 were excluded due to the withdrawal of consent. Eight patients out of 131 experienced two unrelated anaphylactic events during the study period, resulting in 138 anaphylactic events investigated individually. Of the events investigated, 15.94% (n = 22) were biphasic, 5.07% (n = 7) were non-anaphylactic biphasic, and the majority (79.0%, n = 109) were uniphasic (Table 1).

**Table 1:** Cohort demographics

Comparator	Uniphasic (n = 109)	Non-anaphylactic biphasic (n = 7)	Biphasic (n = 22)	*P* value
Age, median y	31.00	20.00	31.50	0.8565
Pediatric cases (< 13 y), No. (%)	9 (8.3%)	–	1 (4.5%)	–
Females, No. (%)	68 (62.4%)	5 (71.4%)	14 (63.6%)	0.8900
History of anaphylaxis, No, (%)	58 (53.2%)	3 (42.9%)	14 (63.6%)	0.5528
History of Asthma, No (%)	43 (39.4%)	3 (42.9%)	7 (31.8%)	0.7483
Time to onset of symptoms for first reaction, minutes	N = 90	N = 6	N = 17	0.9793
Median	10.00	10.00	15.00	
Mean	31.21	21.17	35.82	
Time to resolution of symptoms for first reaction, hours	N = 97	N = 6	N = 19	0.0677
Median	3.750	5.500	2.500	
Mean	6.084	6.250	9.487	
Time to onset of symptoms for second reaction, hours	–	N = 6	N = 17	0.9585
Median		10.10	11.00	
Mean		20.21	19.00	

The median age and sex of patients across anaphylactic events were comparable. Among uniphasic, non-anaphylactic biphasic, and anaphylactic biphasic events, there were no significant differences in prior clinical history of anaphylaxis (p = 0.5528) or asthma (p = 0.7483), time to onset of symptoms for first reactions (p = 0.9793) or time to resolution of symptoms for first reaction (p = 0.0677; uniphasic = 3.75 h, non-anaphylactic biphasic = 5.50 h, biphasic = 2.50 h). The mean recurrence time of a second phase reaction was similar (p = 0.9585) between non-anaphylactic biphasic events (20.2 h) and biphasic events (19.0 h). There was also no correlation between time to resolution and recurrence time in this cohort of biphasic events (r = 0.1217, p = 0.6755).

**Fig. 1:**
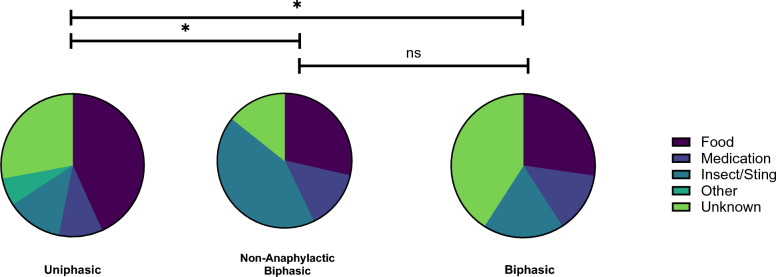
Reaction profiles. The proportion of anaphylactic triggers across the responder groups is represented by pie charts. The reaction profiles significantly differed between uniphasic responders and non-anaphylactic biphasic and biphasic responders (p = 0.0179 and p = 0.0375, 2-way ANOVA with Tukey’s Multiple Comparisons test). Reaction profiles were comparable between non-anaphylactic biphasic and biphasic responders (p = 0.8665, 2-way ANOVA with Tukey’s Multiple Comparisons test)

Food was the leading trigger for uniphasic events (43%, n = 47), whereas insect stings (43%, n = 3) and unknown antigens (41%, n = 9) were the most common triggers for non-anaphylactic biphasic and biphasic events, respectively (Table 2). The distribution of triggering antigens, categorized as food, medication, insect stings, miscellaneous, and unknown, is significantly different (p < 0.05) between uniphasic biphasic reactions (Fig. 1). Anaphylactic and non-anaphylactic biphasic reactions had similar distributions of anaphylactic triggers (p = 0.8655).

**Table 2:** Triggers of anaphylactic events

Categories	Uniphasic No. (% of group)	Non-Anaphylactic Biphasic No. (% of group)	Biphasic No. (% of group)
All food	47 (85.5)	2 (3.6)	6 (10.9)
Egg	3 (6.4)	0 (0)	0 (0)
Sunflower	3 (6.4)	0 (0)	0 (0)
Seafood	4 (8.5)	0 (0)	0 (0)
Shrimp	1 (2.1)	0 (0)	2 (33.3)
Peanut	9 (19.1)	0 (0)	3 (50.0)
Tree nut	10 (21.3)	1 (50.0)	0 (0)
Unknown nuts	2 (4.3)	1 (50.0)	0 (0)
Other foods	15 (31.9)	0 (0)	1 (16.7)
**Overall percentage of food as a trigger**	***43.1%***	***28.6%***	***27.3%***
All medication	11 (73.3)	1 (6.7)	3 (20.0)
Subcutaneous allergy immunotherapy	3 (27.3)	1 (100.0)	0 (0)
NSAIDs	1 (9.1)	0 (0)	0 (0)
Penicillin	1 (9.1)	0 (0)	0 (0)
Lincomycin	0 (0)	0 (0)	1 (33.3)
Glycopeptide	1 (9.1)	0 (0)	0 (0)
Quinolone	1 (9.1)	0 (0)	0 (0)
Sulfonamides	2 (18.2)	0 (0)	0 (0)
Miscellaneous	2 (18.2)	0 (0)	2 (66.7)
**Overall percentage of medications as a trigger**	***10.1%***	***14.3%***	***13.6%***
All insect/sting	14 (66.7)	3 (14.3)	4 (19.0)
**Overall percentage of insect/sting as a trigger**	***12.8%***	***42.9%***	***18.2%***
Miscellaneous/unknown	37 (78.7)	1 (2.1)	9 (19.1)
**Overall percentage of other/unknown as a trigger**	***33.9%***	***14.3%***	***40.9%***

Bold and italicized percentages summarize each subsection of triggers of anaphylactic events

**Fig. 2:**
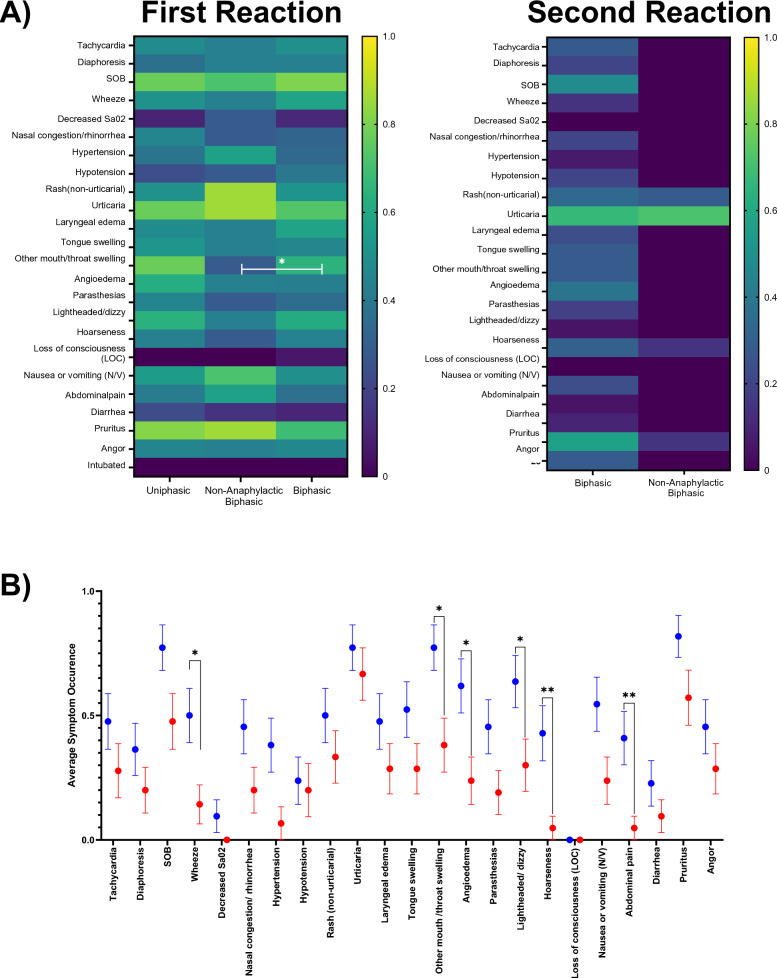
Symptoms profiles. **A** Heat map (left) representing proportional occurrence of specific symptoms across the first anaphylactic response of uniphasic, non-anaphylactic biphasic reaction responders. Biphasic patients were more likely (p = 0.0361, 2-way ANOVA with Tukey’s Multiple Comparisons test) to report “other mouth/throat swelling” than non-anaphylactic biphasics. Heat map (right) representing proportional occurrence of specific symptoms across the second anaphylactic response of non-anaphylactic biphasic and biphasic responders. The secondary anaphylactic reaction of biphasics had significantly less symptoms (p < 0.0001, 2-way ANOVA with Tukey’s Multiple Comparison’s test) than non-anaphylactic biphasics. **C** Graph displaying the proportion of symptoms experienced by biphasic responders comparing their first and second anaphylaxis reactions. There were six symptoms which were significantly decreased in biphasic responders’ second (red) reaction vs first (blue)

The initial presenting symptoms were not significantly different for patients with uniphasic or biphasic reactions (p > 0.9080, Fig. 2A).However, among patients experiencing a biphasic reaction, they were characterized with significantly greater (p < 0.05) “other mouth/throat swelling” symptoms than non-anaphylactic biphasic events. Second reactions were less burdensome in terms of symptoms than the initial anaphylactic event for both biphasic (p = 0.0002) and non-anaphylactic biphasic (p < 0.0001) events. Specifically, the symptoms of “wheeze”, “other mouth/throat swelling”, “angioedema”, “lightheadedness/dizzy”, “hoarseness”, and “abdominal pain” were significantly decreased (p < 0.05) in anaphylactic second reaction events compared to the first (Fig. 2B).

**Fig. 3:**
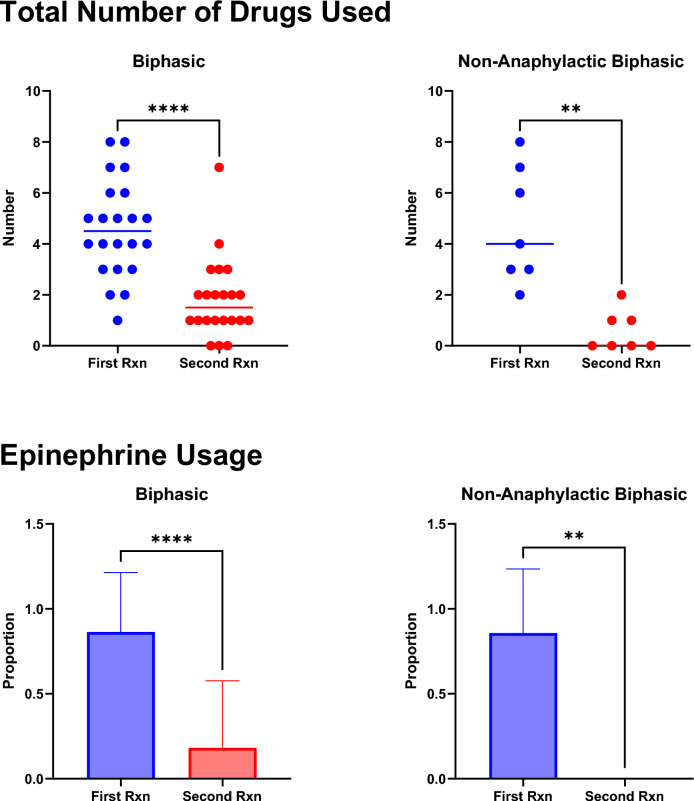
Drugs and Epinephrine Usage. Total Number of Drugs Used (top): The number of drugs used to manage first (red) and second (blue) reactions in biphasic and non-anaphylactic biphasic reactors. For both responder groups, significantly fewer drugs were used to treat their second reaction than the first reaction (p < 0.05, Mann–Whitney test). Epinephrine Usage (bottom): The proportion of non-anaphylactic and biphasic patients who received epinephrine in managing their first and second reactions. For both responder groups, a significantly smaller proportion of patients were administered epinephrine for their second reaction (p < 0.05, Mann–Whitney test)

The management of the first anaphylactic reaction was similar in all groups, with a comparable average number of drugs used, including epinephrine, beta-agonists, histamine (H)1 and H2 antagonists, and corticosteroids (Table 3). Most patients received one or two doses of epinephrine for their first reaction; however, two patients received three and four doses for the biphasic events, respectively. Only four events of biphasic anaphylaxis were treated with epinephrine, all requiring only one dose. The average number of medications used to manage the second reaction for biphasic events was significantly greater than for non-anaphylactic biphasics (p = 0.0289). Compared with the initial reactions, second reactions were generally managed with less medication, including epinephrine (p < 0.005), for both anaphylactic (p < 0.0001) and non-anaphylactic (p < 0.0012) biphasic reactions (Fig. 3).

**Table 3:** Overall drug management of anaphylactic events

Comparator	Uniphasic (n = 109)	Non-anaphylactic biphasic (n = 7)	Biphasic (n = 22)	*P* value
First reaction (n = 138)				
Average number of medications	4.193	4.714	4.591	0.5812
Epinephrine, no. (%)	86 (78.9)	6 (85.7)	19 (86.4)	0.6794
Epinephrine doses administered				
One	71	4	13	
Two	15	2	4	–
Three	0	0	1	
Four	0	0	1	
Average total dose (mg)	0.545	0.600	0.515	0.7678
Epinephrine auto injector, no. (%)	32 (37.2)	2 (33.3)	5 (26.3)	0.7260
H1 antagonist, no. (%)	106 (97.2)	7 (100)	21 (95.5)	0.8081
H2 antagonist, no. (%)	67 (61.5)	4 (57.1)	15 (68.2)	0.8056
Corticosteroid, no. (%)	78 (71.6)	4 (57.1)	16 (72.7)	0.7060
Beta agonist, no. (%)	29 (26.6)	1 (14.3)	3 (13.6)	0.3583
Average total dose (mg nebulized)	*6.39*	*5.00*	*3.93*	*0.9100*
Intravenous fluid (IV), no. (%)	49 (45.0)	4 (57.1)	10 (45.5)	0.8223
IV volume (L)	0.449	0.571	0.454	0.8223
Post-resolution medications, no. (%)	36 (33.0)	2 (28.6)	7 (31.8)	0.9673
Average number of post-resolution drugs	0.449	0.286	0.364	0.8770
Second reaction (n = 29)				
Average number of medications	–	0.57	1.82	**0.0289**
Epinephrine used, no. (%)	–	0 (0)	4 (18.2)	–
Epinephrine doses administered				
One			4	
Two	–	–	0	–
Three			0	
Four			0	
Average total dose (mg)	–	–	0.45	–
H1 antagonist, no. (%)	–	3 (42.9)	16 (72.7)	0.1929
H2 antagonist, no. (%)	–	0 (0)	3 (13.6)	–
Corticosteroid, no. (%)	–	0 (0)	6 (27.3)	0.3866
Beta agonist, no. (%)	–	0 (0)	2 (9.1)	–
Average total dose (mg nebulized)	–	–	2.7	–
Intravenous fluid (IV), no. (%)	–	0 (0)	2 (9.1)	–
IV volume (L)	–	–	0.05	–

Bold values are statistically significant (p<0.05)

**Fig. 4:**
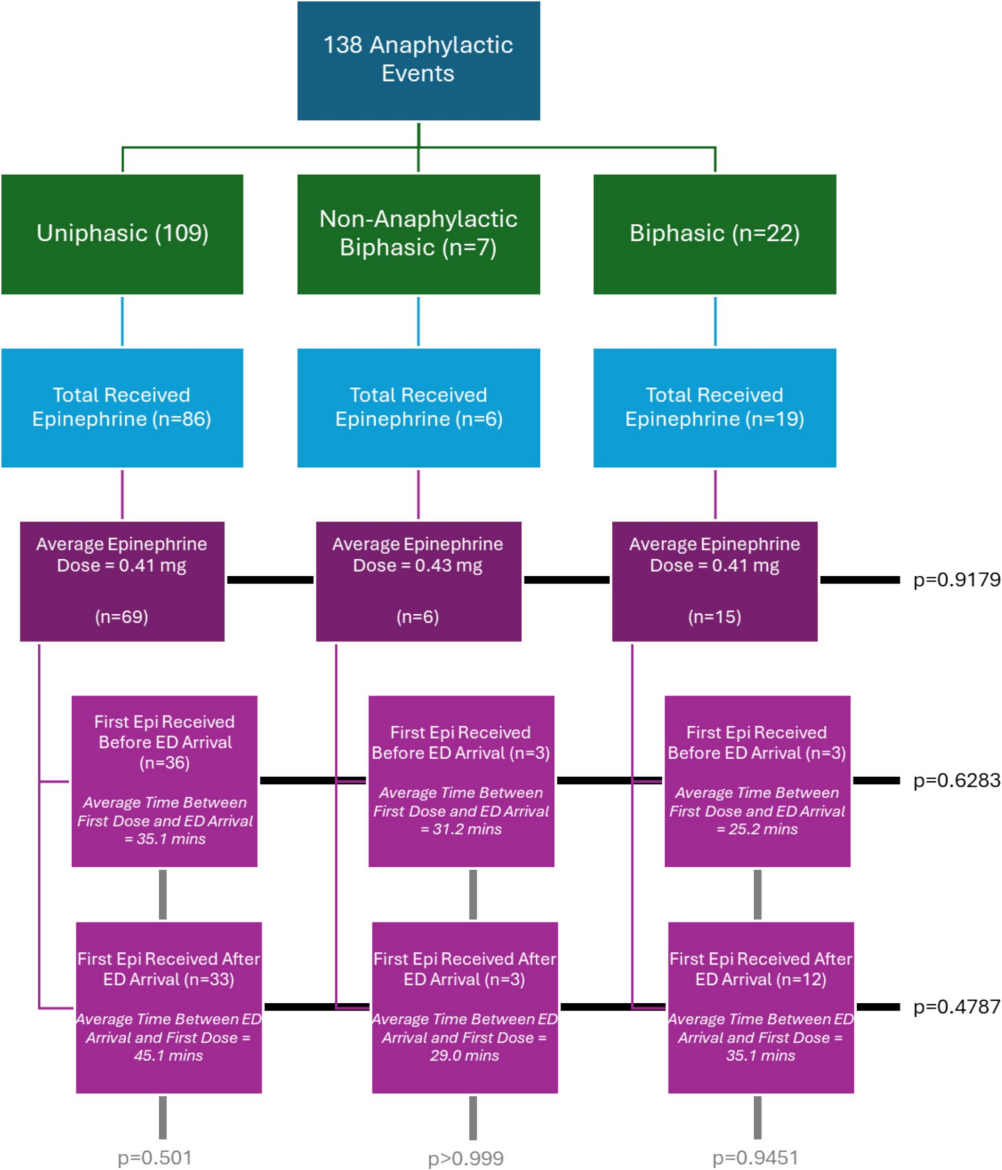
Flowchart depicting Epinephrine Usage, Doses, and Timings. 138 anaphylactic events were sub-categorized into uniphasic, non-anaphylactic biphasic, and biphasic events. Patients who received epinephrine in each group were identified, and sub-analyses of epinephrine dosage and timing of dose administration relative to ED arrival were conducted. No significant differences were found between the first epinephrine dosage across each responder group (p = 0.9179, Kruskall-Wallis test). Among patients who received their first dose of epinephrine before ED arrival, there was no significant difference in the time between the first dose administration and ED arrival for uniphasic, non-anaphylactic biphasic, and biphasic responders (p = 0.6283, Kruskall-Wallis test). Similarly, among patients who received their first dose of epinephrine after ED arrival, there was no significant difference in the time between ED arrival and the first dose administration for uniphasic, non-anaphylactic biphasic, and biphasic responders (p = 0.4787, Kruskall-Wallis test). The timing of epinephrine administration and ED arrival was comparable among the responder groups (p > 0.05, Mann–Whitney test)

In this cohort, 80% of cases (n = 111) were treated with at least one dose of epinephrine in response to their first anaphylactic event (Fig. 4). The exact time and dose of epinephrine used was known and reported for 90 cases by either the ED, Emergency Medical Services (EMS) or by the patient themselves. There was no significant difference in the average epinephrine dose administered to patients experiencing an initial anaphylactic event (p = 0.9179; Fig. 4). Overall, 46.7% (n = 42) received their first epinephrine dose before arriving in the ED, and 53.3% (n = 48) received their first epinephrine dose after arriving in the ED (Fig. 4). There was no significant difference between the responder groups (e.g., uniphasic vs non-anaphylactic biphasic vs biphasic) groups (p > 0.05) in the mean time between first dose of epinephrine and ED arrival. Within each of the responder groups, there was no significant difference in the time between epinephrine administration and ED arrival for those patients who received epinephrine before ED arrival or after (p > 0.05). The average time to first epinephrine treatment before ED arrival was 30.5 min across groups and 36.4 min when received after ED arrival (Fig. 4).

## Discussion

### Interpretation of Findings

Biphasic anaphylaxis has a wide range of reported incidences, and in our current study, it was 16%. tThe mean time of second phase reaction was 19.0 h after the first reaction. However, it is relevant to recognize the lack of significant difference between the timing of recurrence between biphasic and non-anaphylactic biphasic events (19.0 vs 20.2 h) as well as the number of second phase reactions that started in less than 8 h (31.8% vs 28.6%). Second reactions had less severe symptom profiles and a correspondingly lower number of medications used for both anaphylactic and non-biphasic events. We found no significant differences in epinephrine administration between all three types of responders, including timing related to ED arrival. The time for the administration of a first dose of epinephrine was comparable whether it was received before or after ED arrival (between 30 and 37 min). The time of first epinephrine administration once a patient arrived in ED at our site was comparable to other clinical practices. Cha et al. reported that epinephrine was administered within 30 min of hospital arrival for 52.6% of patients, while the remaining 47.4% received epinephrine after 30 min or more [18]. There are many factors that may influence the speed of first epinephrine administration, both before and after hospital admission, which are not captured in our study, though they warrant a closer examination.

Interestingly, most biphasic events (80.0%) received a first dose of epinephrine after ED arrival and were more likely to receive subsequent doses, suggesting that delayed administration of epinephrine may have played a role in biphasic anaphylaxis. We did find a statistically significant difference in the distribution of antigens that caused anaphylaxis between the uniphasic with non-anaphylactic biphasic events responders (p = 0.0179) and anaphylactic biphasic responders (p = 0.0375). More specifically, 41% of patients who developed biphasic anaphylaxis had reactions to an unknown trigger (n = 9). This is in keeping with a study that identified unknown trigger as a risk for biphasic reactions [19]. Kraft et al. used data from the European Anaphylaxis Registry to analyze possible risk factors of biphasic anaphylaxis. They analyzed a cohort of 435 cases of biphasic anaphylaxis and compared them with 8736 uniphasic reactions. The authors also saw no difference between the main groups of elicitors of anaphylaxis such as food, drugs and insects. However, they also saw that peanut, and to a lesser extent tree nut, was associated with a higher rate of biphasic anaphylaxis; 9.6% of the reactions were biphasic [20].

### Comparison to Previous Studies

In our 2007 study, the incidence of biphasic reactions in Kingston, Ontario, Canada, was 19%, compared to 16% in our current study. The mean time for the occurrence of the second-phase reaction was 19.0 h (range 0.5–72 h), which is longer than the previously reported window of 10 h (range 2–38 h) [17]. Only 31.8% of biphasic events started the second phase less than 8 h after the initial reaction. This supports the results from a study where the interval for the second phase ranged from 4.5 to 29.50 h and another by Lee et al., which reported that approximately half of biphasic reactions occur within 6–12 h [19, 21]. Lieberman et al. also suggested this, concluding that second-phase reactions can occur up to 72 h following the initial reaction [22]. Our study did report a higher incidence of biphasic anaphylaxis compared to other studies, such as the 2019 meta-analysis by Kim et al. (6.5%) and the 2020 systematic review by Chu et al. (3.65%), which is likely a reflection of the inherent sample bias from our single-center study [23].

The risk factors for developing biphasic anaphylactic reactions remain unclear. Previous studies showed that delayed or repeated administration of epinephrine is associated with biphasic reactions [24]. Our study found that 80.0% of patients experiencing a biphasic reaction had their first dose of epinephrine in the ED and 31.6% of biphasic responders required subsequent doses of epinephrine. This finding is consistent with the findings of Liu et al. who found that 19% of patients with biphasic reactions required two doses of epinephrine. This group also found that a biphasic course was associated with an ED setting of the first epinephrine dose (OR 3.72; 95% CI 1.36–10.14) [25].

Rapid and complete resolution of acute symptoms has also been related to a decreased risk of developing biphasic reactions [16, 17]. In this study, we did not find a correlation between time to resolution and recurrence time in this cohort of biphasic events (r = 0.1217, p = 0.6755). On average, initial anaphylactic events were resolved within 6 h for uniphasic and non-anaphylactic biphasic events whereas biphasic events took longer, at 9.5 h. This finding conflicts with our 2007 report, whereby the biphasic reactors, however, took significantly longer to resolve their initial symptoms than uniphasic reactors (133 vs 112 min; *P* = 0.03) [17].

We also examined the role of corticosteroids; theoretically, their anti-inflammatory and gene expression modulation properties counteract the process of anaphylaxis, though systematic reviews of the literature do not support their role in preventing biphasic responses even as an adjunctive therapy [2, 26]. In our cohort, similar rates of corticosteroid usage were observed between all groups. This appears to be consistent with the literature suggesting that corticosteroids do not prevent biphasic anaphylaxis [2, 27].

### Strengths and Limitations

In our study, the timing of biphasic symptoms relied on a chart review with retrospective patient-reported data. Therefore, the consistency of the data may be limited, and recall bias may impact the symptoms reported by the participants. However, clinical definitions were used to identify patients with anaphylaxis. Another weakness is the limited scope of the data, as only a single centre was assessed. While our ED serves patients in Kingston and the surrounding area, our findings may be limited to our own tertiary care centre and the region it serves. Further, this study could not assess anaphylactic reactions that occurred in the community that were not treated by the ED, and so the results of this study are potentially biased by selection of severe anaphylaxis that would present to the ED. This study did not directly assess the impact of epinephrine administration timing. A surrogate for those data was whether epinephrine was administered pre- or post-ED arrival, but high-quality evidence on outcomes when epinephrine is given within 30 min after onset of symptoms is desperately needed. Finally, given that almost half of the patients who had biphasic reactions were related to unknown triggers, there is a possibility that patients were re-exposed to their trigger and subsequently developed a second uniphasic anaphylactic reaction.

### Research and Clinical Implications

The mean length of time between reactions in biphasic anaphylaxis, shown in this study to be 19.0 h, raises concerns about prolonging the monitoring period following primary reactions up to 10 h compared to the current usual monitoring period between 4 and 8 h. Extending observation periods can be difficult to achieve in busy EDs and may not be economically advantageous in some circumstances (e.g., resolved anaphylaxis with low risk of biphasic anaphylaxis), and so alternative solutions should be considered, such as patient education, facilitating access to emergent medical care, and prescribing an epinephrine autoinjector at discharge [28]. Similarly, our study noted that most biphasic responders received their first dose of epinephrine after arriving at the ED. However, the reasons for their delay in administration and associated circumstances require further inquiry.

## Conclusion

Biphasic reactions remain poorly defined regarding incidence, features, and predictors. Our study, with its specific population of patients from Kingston, ON, showed that biphasic anaphylaxis occurred in 16% of patients with anaphylaxis reporting to the ED. The onset of the second phase reaction was longer than expected, with an average of 19 h, with only 31.8% experiencing symptoms less than 8 h following the initial reaction. Patients with biphasic reactions experienced a prolonged duration of symptoms following their initial reaction. Further larger studies are needed to explore possible predictors and the relation of delayed epinephrine and corticosteroid administration with the occurrence of second-phase reactions.

## Data Availability

The datasets generated and analysed during the current study are not publicly available but are available from the corresponding author on reasonable request.

## Abbreviations

ICON: International Consensus on Anaphylaxis
Ig: Immunoglobulin
ED: Emergency department
HSREB: Health Sciences Human Research Ethics Board
CPSP: Canadian Pediatric Surveillance Program
EMS: Emergency Medical Service
H: Histamine
